# Case report: Retroperitoneal solid pseudopapillary neoplasm associated with multiple hepatic metastases

**DOI:** 10.3389/fonc.2024.1335930

**Published:** 2024-01-30

**Authors:** Lin Chen, Mengchen Yuan, Meng Wang, Chenglong Luo, Mengyu Gao, Qingbo Huang, Zhenqian Li, Zhigang Zhou

**Affiliations:** ^1^ Department of Radiology, The First Affiliated Hospital of Zhengzhou University, Zhengzhou, China; ^2^ Department of Pathology, The First Affiliated Hospital of Zhengzhou University, Zhengzhou, China

**Keywords:** retroperitoneal neoplasm, hepatic metastasis, abdominal neoplasm, extrapancreatic, solid pseudopapillary neoplasm

## Abstract

Solid pseudopapillary neoplasm (SPN) is a rare tumor mostly occurring in the pancreas. They are low-grade malignant tumors of the exocrine pancreas that occasionally metastasize, usually to the liver or peritoneum. Additionally, multiple metastases of extrapancreatic SPN to the liver are extremely rare and have been reported before. This study presents a case of a 13-year-old male patient with retroperitoneal SPN and multiple hepatic metastases. The patient presented with abdominal trauma and underwent enhanced CT, which revealed upper pancreatic occupancy and three hypodense foci in the right lobe of the liver. Moreover, increased spleen size was noted. The patient’s serum tumor marker CA125 was increased to 39.00 U/mL (*N* < 35.0 U/mL), and circulating tumor cells were elevated to 10.2 FU/3 mL (*N* < 8.7 FU/3 mL). The patient underwent retroperitoneal occupancy resection and splenectomy, followed by resection of liver metastases 7 months after the surgery. Furthermore, multiple liver metastases from retroperitoneal SPN were confirmed postoperatively. The patient recovered for 1 year without tumor recurrence. This case emphasizes the importance of evaluating serum tumor markers and medical imaging in young patients as well as the fact that surgery appears to be the preferred treatment option for multiple metastases in SPN.

## 1 Introduction

In 1959, Frantz first described solid pseudopapillary neoplasm (SPN) as a rare low-grade malignant tumor (1). The tissue origin and pathogenesis of solid pseudopapillary pancreatic neoplasm are unknown. Only 3% of all pancreatic tumors are of this type (2). SPN is a type of epithelial tumor made up of loosely packed polygonal cells. These cells surround fragile blood vessels and form solid masses and pseudopapillary structures formed by morphologically uniform cells; hemorrhage and cystic degeneration are also observed. SPN is most common in young female patients. The median age is 20–40 years, and the male-to-female ratio is 1:10 (3). SPNs can occur in any part of the pancreas, but extrapancreatic SPNs are extremely rare. They can be easily misdiagnosed because most patients do not have specific clinical signs or symptoms. Metastasis is very rare, and its most common site is the liver.

## 2 Case description

**Figure 1:**
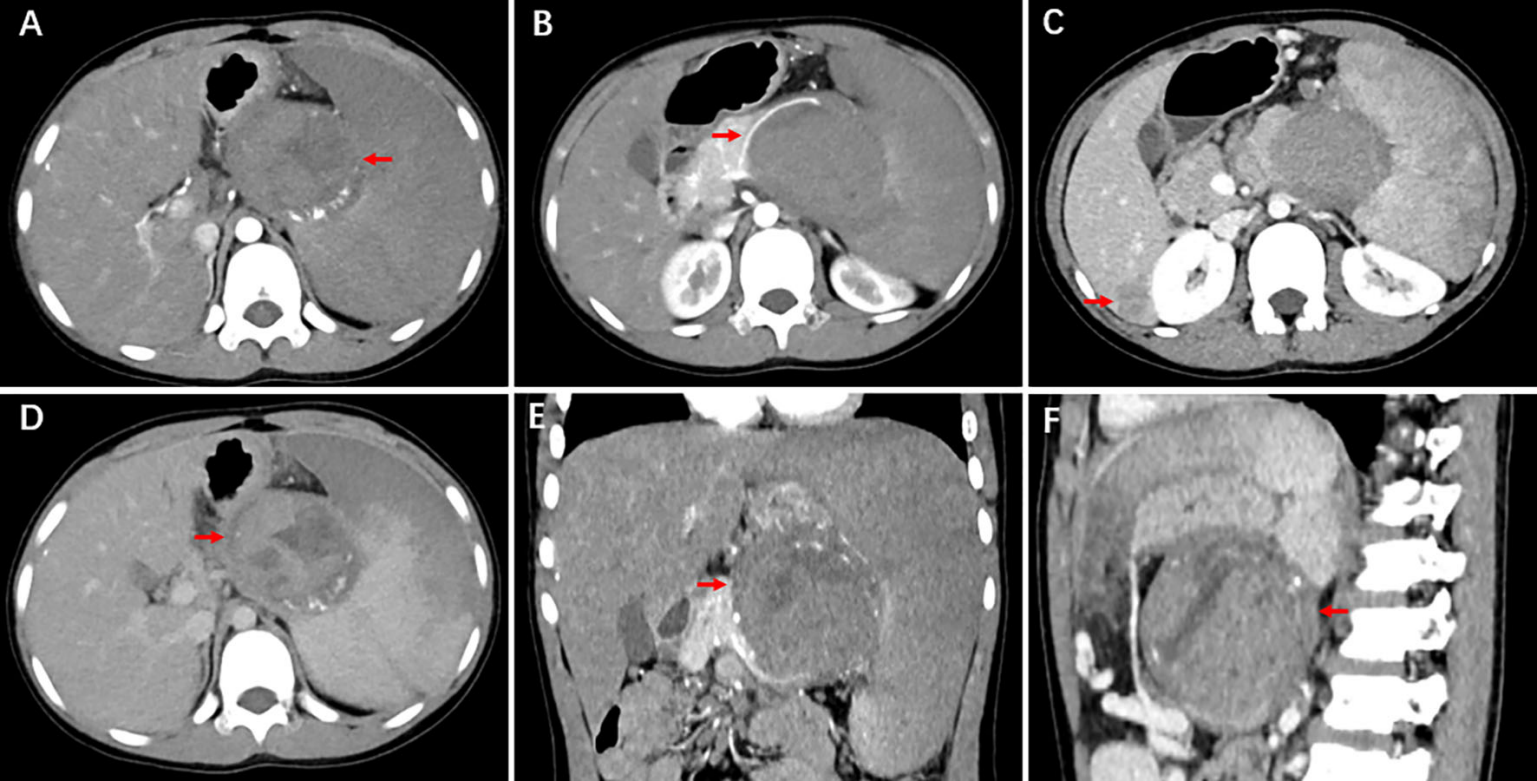
CT shows upper pancreatic occupancy and three hypodense foci in the right lobe of the liver. A round cystic-solid mass is observed at the superior border of the pancreas with heterogeneous internal density. Arcuate calcifications and tortuous vascular are seen at mass margins. It has a relatively well-defined border. The enhancement scan shows progressive enhancement, with solid and envelope enhancing and cystic areas not enhancing. The mass is well demarcated from the spleen, and the splenic artery is displaced by compression.

A 13-year-old male patient was referred to the First Affiliated Hospital of Zhengzhou University. A CT examination for abdominal trauma revealed retroperitoneal occupancy. The patient presented with abdominal pain after falling 4 days earlier. The pain spread gradually to the entire abdomen. It was persistent and increased in paroxysms. It was accompanied by nausea, vomiting, and loss of appetite. Physical examination revealed mild abdominal distension, slightly distended left upper abdomen, scattered skin abrasions, and pressure and rebounding pain throughout the abdomen. The spleen was enlarged; the lower border was 1 cm above the umbilicus and the right side to the anterior midline. The results of laboratory tests were as follows: neutrophils, 6.92 × 109/L; C-reactive protein, 58.48 mg/L; tumor-associated antigen 125, 39.00 U/mL; non-small-cell lung cancer antigen 21-1, 3.62 ng/mL (*N* < 3.3); neuron-specific enolase, 25.20 ng/mL (*N* < 25); and elevated circulating tumor cells, 10.2 FU/3 mL (*N* < 8.7). Direct-enhanced CT of the abdomen revealed the following: round cystic-solid mass at the superior border of the pancreas with heterogeneous internal density. Arcuate calcifications and tortuous vascular are detected at mass margins. The mass measured about 7.2 × 6.4 × 7.1 cm. It had a relatively well-defined border. The enhancement scan showed progressive enhancement, with solid and envelope enhancing and cystic areas not improving. The mass is well demarcated from the spleen, and the splenic artery is displaced by compression (**Figure 1**). The body of the pancreas is displaced downward by compression of the tail. No dilatation of the pancreatic duct was observed, and slightly larger lymph nodes were detected around the pancreas. Three hypodense foci were seen in the right liver lobe measuring 3.0 × 2.4 cm, 2.5 × 2.0 cm, and 0.2 × 0.2 cm. They were isointense during the delay period. Wedge-shaped and linear hypodense structures were present within the spleen. The ultrasound showed an enlarged spleen with heterogeneous echogenicity of the parenchyma. Mixed echoes were detected at the splenic hilum with clear, regular borders. There was a peripheral punctate blood flow signal. Based on the comprehensive analysis of imaging studies and corresponding clinical observations, our initial diagnostic considerations were splenic infarction, SPN, or pancreatoblastoma. Furthermore, the possibility of multiple metastases in the right lobe of the liver is under consideration.

**Figure 2:**
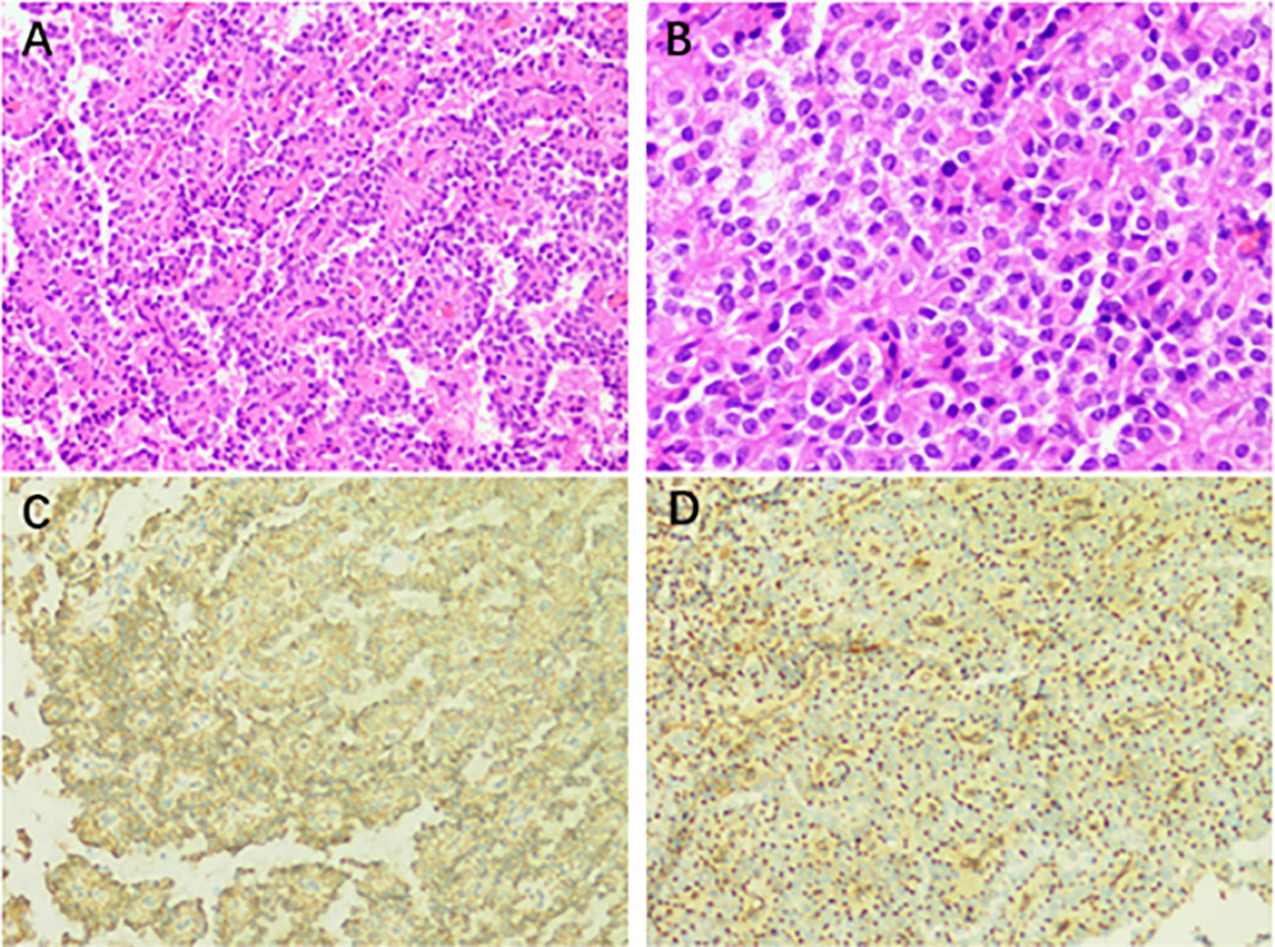
**(A)** Postoperative pathological result of the retroperitoneal occupancy: retroperitoneal solid pseudopapillary neoplasm (H&E staining, ×200). **(B)** The tumor cells have a mild morphology. They are oval, with medium cytoplasm and common nuclear grooves (H&E staining, ×400). **(C)** Immunostaining for β-catenin showing a positive reaction (×200). **(D)** Immunostaining for CD99 displaying a positive reaction (×200).

The patient was scheduled for retroperitoneal lesion resection and splenectomy. During surgery, the pancreas was clearly separated from the tumor. The mass is primarily supplied by a branch of the splenic artery. Intraoperatively, the spleen was visibly enlarged, and the upper pole was dark red. The rest of the spleen was dark purple with a tendency to blacken. A grayish-white mass was visible from the upper edge of the pancreas to the lower part of the diaphragm, just below the hilum of the spleen. The mass was well demarcated and measured about 8.2 × 7.5 × 7.3 cm. It was hard and poorly mobile. The splenic artery passed through the upper pole of the mass. During this surgery, the tumor was completely removed from the retroperitoneum. The wall of the mass contains calcifications. It was well separated from the adjacent pancreatic parenchyma without invasion. Microscopically, a pseudopapillary structure was formed around the axis of the blood vessels via uniform individual tumor cells. The immunohistochemical analysis revealed that the tissue was positive for AE1/AE3 (CK), CK8/18, Syn, CgA, β-catenin, CD56, CD99, and Ki-67 (approximately 2%+) (**Figure 2**). Based on the immunohistochemistry and history, the final diagnosis was chronically bruised splenomegaly with retroperitoneal SPN. After symptomatic treatment, the patient was discharged from the hospital with postoperative platelet elevation.

At 7 months postoperatively, the patient was revisited for an enhanced abdominal CT. A CT scan of the abdomen displayed three irregularly shaped, well-defined wedge-shaped lesions of low density in the liver. The mass intensified markedly in the arterial phase and decreased unevenly in the venous phase with circumferential enhancement. We synthesized it to look SPN-metastasized. The patient underwent surgical resection of all three liver masses. A biopsy of enlarged abdominal lymph nodes was performed. Operative findings included mass in liver segments V, VI, and VII measuring 0.2 × 0.2 cm, 3 × 2.5 cm, and 2.5 × 2.0 cm, respectively. The mass is grayish-white and hard in texture. The immunohistochemical analysis showed that the tissue was positive for CK8/18, Syn,β-catenin, CD10, CD56, CD99, AAT, and Ki-67 (approximately 5%+) and negative for CgA, hepatocyte, and arginase. Multiple SPN metastases in the liver were identified using immunohistochemistry and medical history. At the last follow-up, the patient was clinically asymptomatic, and no liver or abdominal lesions were detected on abdominal CT at 12 months after the first surgery.

## 3 Discussion

SPN is a rare low-grade malignant exocrine tumor of the pancreas, also known as solid papillary epithelial tumor, papillary cystic tumor, solid cystic tumor, and Frantz tumor. SPN pathogenesis remains unclear. It could be related to mutations in the β-catenin gene in the WNT signaling pathway. Mutations in the exons result in impaired β-catenin phosphorylation, causing the activation of oncogenes and disease development (4). Currently, the widely accepted theory is that SPN originates from a type of pluripotent cell with multiple differentiation potentials. It could present in infancy and early childhood and be discovered after years of growth (5). Our patient’s young age, large tumor size, and lack of clinical symptoms are consistent with the hypothesis description.

With the number of reports related to this disease gradually increasing, there is a more comprehensive understanding of both the imaging and histologic features of the disease. SPN is most prevalent in young women. It is solitary and is most often located in the tail of the pancreas, but it can also occur outside the pancreas. This tumor is a pancreatic borderline tumor. Moreover, extrapancreatic metastasis has occurred in approximately 10%–15% of cases at the time of presentation. High malignant potential is suggested when the tumor is >5 cm (6), is lobulated or with a discontinuous envelope (7), has peripheral vascular infiltration, is hepatic, or has lymph node metastases and when imaging shows dilated pancreatic ducts (8). In this case, the mass was 8.2 cm in length. It had liver metastases and enlarged lymph nodes, which were highly malignant.

Most clinical manifestations of the disease are subtle and are typically detected incidentally on imaging, and CT is the main preoperative diagnostic modality for SPN. The SPN pathology is highly distinctive, with uniform, single tumor cells forming a pseudopapillary structure around the vascular axis (9). Immunohistochemistry revealed varied expressions of AAT, Vim, β-catenin, Syn, neuron-specific enolase, CD10, and CD56. If the Vim and β-catenin nuclei are positive, the diagnosis of the disease is likely. The immunohistochemical findings in this case align with this conclusion.

There are currently few reports on the imaging of extrapancreatic SPN. On imaging, pancreatic SPN typically appears as a cystic, solid mass with exophytic growth. The envelope is complete, the edges are clearly defined, and the calcifications can be seen. The solid portion has slightly less progressive enhancement than normal pancreatic tissue. The mass envelope has been improved (10). The “floating cloud sign” is visible in patients who are predominantly cystic or have similar proportions of cystic-solid mass. Eggshell-like calcifications are a typical manifestation of this sign. Miyazaki reported the first SPN case that originated in the retroperitoneum without an ectopic pancreas. Enhanced CT of the abdomen showed a cystic, solid mass visible in the left adrenal region with an envelope and clear borders, and eggshell-like calcifications were also detected (11). In this case, the patient presented with abdominal trauma and developed splenic infarction. CT revealed a retroperitoneal space-occupying lesion. The lesion was compressing the splenic artery. The patient was young and male, and we initially suspected that the patient had a pancreatoblastoma or solid pseudopapillary tumor. Prior studies reported that the liver is the most common organ for SPN metastasis. Tumor cells can metastasize to the liver through the superior mesenteric and portal veins (12). Liver metastases from SPN can be detected at initial diagnosis or postoperatively. Their imaging presentation is similar to that of the primary focus. However, SPN progresses relatively slowly. Metastatic foci are most often associated with cystic changes. They are characterized by the growth of cystic solid masses with pseudopapillary structures (13). In this case, the metastases were cystic and solid. The pathologic findings demonstrated pseudopapillary growth, which is consistent with the literature.

It remains difficult to diagnose extrapancreatic SPN accurately preoperatively. However, the presence of cystic solid masses and cystic masses with eggshell-like calcifications at least suggests the diagnosis. MRI is better than CT in detecting the cystic or solid components of the tumor. Moreover, MRI has specific imaging features that are reliably correlated with clinicopathological features (14). We will continue to explore the learning of MRI for SPN. The diagnostic specificity of the disease can be enhanced by recognizing the rare possibility of extrapancreatic primary SPN. Furthermore, endoscopic ultrasound-guided fine-needle biopsy (EUS-FNB) has become an important tool for diagnosing pancreatic and nonpancreatic lesions with high sensitivity and specificity (15). However, because the patient was referred to our hospital for trauma and later developed a large splenic infarction, EUS-FNB was not performed in this case.

Surgery is the preferred treatment for SPN. Patients who have had surgery have a good prognosis. Even with local infiltration or distant metastasis, long-term survival is possible after surgery (16). Some patients may benefit from other treatments, such as chemotherapy, alcohol injections, transcatheter arterial chemoembolization, radiation therapy, and neoadjuvant chemotherapy for liver transplantation (17). BKP Goh suggested that, for SPN patients with liver metastases, if the metastases are resected, the prognosis is the same as that for SPN without metastases (18). Despite our current understanding of SPNs, research into the prevention, prediction, and treatment of SPN liver metastases is needed.

## 4 Conclusion

**Figure 3:**
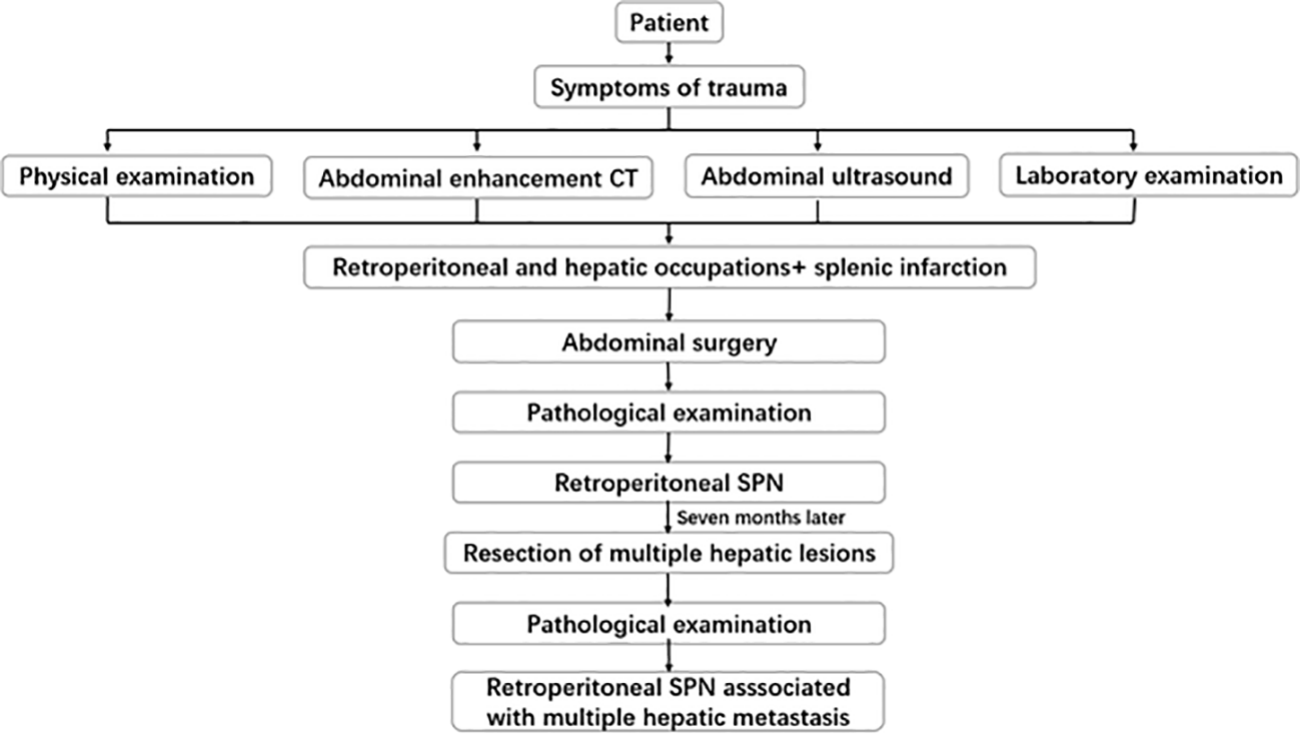
The process of the patient’s visit.

In this study, a rare case of retroperitoneal solid pseudopapillary neoplasm associated with multiple hepatic metastases was reported (**Figure 3**). The SPN diagnosis was based primarily on histopathology and genetic testing. The blood supply of the mass on imaging, as well as its association with peripheral signs, aids in clinical diagnosis and treatment decisions. This is important for differentiating between tumors. This study has the potential to provide useful information for clinical diagnosis and patient follow-up.

## Data availability statement

The original contributions presented in the study are included in the article/supplementary material. Further inquiries can be directed to the corresponding author.

## Ethics statement

The studies involving humans were approved by The First Affiliated Hospital of Zhengzhou University, Zhengzhou, China. The studies were conducted in accordance with the local legislation and institutional requirements. Written informed consent was obtained from the participant/patient(s)' guardian for the publication of this case report.

## Author contributions

LC: Conceptualization, Data curation, Investigation, Resources, Supervision, Validation, Visualization, Writing – original draft, Writing – review & editing. MY: Conceptualization, Investigation, Resources, Supervision, Validation, Writing – original draft, Writing – review & editing. MW: Writing – review & editing. CL: Writing – review & editing. MG: Writing – review & editing. QH: Writing – review & editing. ZL: Writing – review & editing. ZZ: Conceptualization, Data curation, Investigation, Methodology, Project administration, Resources, Validation, Visualization, Writing – original draft, Writing – review & editing.

## References

[1] Frantz VK. Tumors of the pancreas. In: Atlas of Tumor Pathology, 1st edition. Washington DC, USA: US Armed Forces Institute of Pathology (1959). p. 32–3.

[2] Elta GH Enestvedt BK Sauer BG Lennon AM. ACG clinical guideline: diagnosis and management of pancreatic cysts. Am J Gastroenterol (2018) 113:464–79. doi: 10.1038/ajg.2018.14 29485131

[3] Yao J Song H. A review of clinicopathological characteristics and treatment of solid pseudopapillary tumor of the pancreas with 2450 cases in chinese population. BioMed Res Int (2020) 2020:1–11. doi: 10.1155/2020/2829647 PMC7352122 32685461

[4] Wang J Gerrard G Poskitt B Dawson K Trivedi P Foroni L. Targeted next generation sequencing of pancreatic solid pseudopapillary neoplasms show mutations in Wnt signaling pathway genes. Pathol Int (2019) 69:193–201. doi: 10.1111/pin.12778 30811747

[5] Vargas-Serrano B Domínguez-Ferreras E Chinchón-Espino D. Four cases of solid pseudopapillary tumors of pancreas: imaging findings and pathological correlations. Eur J Radiol (2006) 58:132–9. doi: 10.1016/j.ejrad.2005.11.014 16377114

[6] Kang CM Kim HG Kim KS Choi JS Lee WJ Kim BR. Laparoscopic distal pancreatectomy for solid pseudopapillary neoplasm of the pancreas-report of two cases. Hepatogastroenterology (2007) 54:1053–6. 17629037

[7] Chung YE Kim M-J Choi J-Y Lim JS Hong H-S Kim YC. Differentiation of benign and Malignant solid pseudopapillary neoplasms of the pancreas. J Comput Assisted Tomography (2009) 33:689–94. doi: 10.1097/RCT.0b013e31818f2a74 19820493

[8] Kang CM Choi SH Kim SC Lee WJ Choi DW Kim SW. Predicting recurrence of pancreatic solid pseudopapillary tumors after surgical resection: A multicenter analysis in Korea. Ann Surg (2014) 260:348–55. doi: 10.1097/SLA.0000000000000583 24743622

[9] Dinarvand P Lai J. Solid pseudopapillary neoplasm of the pancreas: A rare entity with unique features. Arch Pathol Lab Med (2017) 141:990–5. doi: 10.5858/arpa.2016-0322-RS 28661210

[10] Hu S Lin X Song Q Chen K. Solid pseudopapillary tumour of the pancreas in children: clinical and computed tomography manifestation. Radiol Med (2012) 117:1242–9. doi: 10.1007/s11547-012-0854-2 22744358

[11] Miyazaki Y Miyajima A Maeda T Yuge K Hasegawa M Kosaka T. Extrapancreatic solid pseudopapillary tumor: case report and review of the literature. Int J Clin Oncol (2012) 17:165–8. doi: 10.1007/s10147-011-0261-z 21656203

[12] Naar L Spanomichou D-A Mastoraki A Smyrniotis V Arkadopoulos N. Solid pseudopapillary neoplasms of the pancreas: A surgical and genetic enigma. World J Surg (2017) 41:1871–81. doi: 10.1007/s00268-017-3921-y 28251269

[13] Yang F Jin C Fu D. Evolution of liver metastasis from solid pseudopapillary tumor of the pancreas. Surgery (2017) 161:1739–40. doi: 10.1016/j.surg.2016.05.001 27302102

[14] Yu C-C Tseng J-H Yeh C-N Hwang T-L Jan Y-Y. Clinicopathological study of solid and pseudopapillary tumor of pancreas: emphasis on magnetic resonance imaging findings. World J Gastroenterol (2007) 13:1811–5. doi: 10.3748/wjg.v13.i12.1811 PMC4149957 17465471

[15] Crinò SF Conti Bellocchi MC Di Mitri R Inzani F Rimbaş M Lisotti A. Wet-suction versus slow-pull technique for endoscopic ultrasound-guided fine-needle biopsy: a multicenter, randomized, crossover trial. Endoscopy (2023) 55:225–34. doi: 10.1055/a-1915-1812 35915956

[16] Zhan H Cheng Y Wang L Su P Zhong N Zhang Z. Clinicopathological features and treatment outcomes of solid pseudopapillary neoplasms of the pancreas: A 10-year case series from a single center. J Laparoendosc Adv Surg Tech A (2019) 29:600–7. doi: 10.1089/lap.2018.0704 30741591

[17] Gomez P Yorke R Ayala AG Ro JY. Solid-pseudopapillary neoplasm of pancreas with long delayed liver metastasis. Ann Diagn Pathol (2012) 16:380–4. doi: 10.1016/j.anndiagpath.2011.02.008 21641841

[18] Goh BKP Tan Y-M Cheow P-C Chung AY-F Chow PKH Wong W-K. Solid pseudopapillary neoplasms of the pancreas: an updated experience. J Surg Oncol (2007) 95:640–4. doi: 10.1002/jso.20735 17477365

